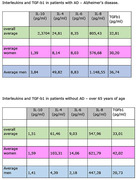# Assessment of inflammatory biomarkers in Alzheimer's disease

**DOI:** 10.1002/alz.084956

**Published:** 2025-01-09

**Authors:** Gustavo A A Santos

**Affiliations:** ^1^ UNICAMP, Campinas, Brazil; Sao Leopoldo Mandic Araras School of Medicine, Araras, Brazil; USP ‐ University of Sao Paulo, Ribeirao Preto Brazil

## Abstract

**Background:**

Neuroinflammation can be considered a risk factor for the onset or progression of Alzheimer's dementia. In a neuroinflammatory process, the death of neurons may accelerate, favoring the progression of Alzheimer's disease. The release of pro‐inflammatory proteins can, for example, cause synaptic dysfunction and impede neurogenesis.

**Method:**

76 participants were invited to participate in this experiment, arranged as follows: Elderly Group without AD: 26 cognitively healthy elderly individuals without a diagnosis of AD, aged 65 years or over. Adults without AD group: 25 adult and non‐elderly individuals, cognitively healthy and without a diagnosis of AD, aged between 19 and 59 years old. Elderly group with AD: 25 patients diagnosed with probable AD, aged 65 years or over. The concentrations of cytokines (IL‐4, IL‐6, IL‐8, IL‐10 and TGF‐β) present in saliva were measured through enzymatic assays (ELISA), using the following kits, respectively: IL‐4, IL‐6, IL‐8, IL‐10 ‐ HCYTOMAG‐60K‐04 CYTOKINE PANEL WITH 04 PLEX (IL4, IL6, IL8 AND IL10); E‐EL‐H0110 ‐ Human TGF‐1(Transforming Growth Factor Beta 1), ELISA Kit

**Result:**

We identified important changes in IL‐4, IL‐6, IL‐8 and TGFb1, with increased expression in participants with AD and a reduction in healthy ones, except for TGFb1, which had exactly the same levels found in participants without AD, except for women, where it was increased, a fact to be investigated

**Conclusion:**

For IL‐10 Cytokines we did not find any type of variation, however IL‐8 was elevated in patients with AD and reduced in patients without AD; IL‐6, IL‐4 and TGF‐b are also elevated in AD patients and reduced in healthy subjects. These findings support the hypothesis that AD is an inflammatory process.